# Consequences of paternally inherited effects on the genetic evaluation of maternal effects

**DOI:** 10.1186/s12711-015-0141-5

**Published:** 2015-08-13

**Authors:** Luis Varona, Sebastián Munilla, Joaquim Casellas, Carlos Moreno, Juan Altarriba

**Affiliations:** Unidad de Genética Cuantitativa y Mejora Animal, Universidad de Zaragoza, 50013 Zaragoza, Spain; Instituto Agroalimentario de Aragón (IA2), Universidad de Zaragoza, 50013 Zaragoza, Spain; Departamento de Producción Animal, Facultad de Agronomía, Universidad de Buenos Aires, 1417 Ciudad Autónoma de Buenos Aires, Argentina; Grup de Recerca en Remugants, Departament de Ciència Animal i dels Aliments, Universitat Autònoma de Barcelona, 08193 Bellaterra, Spain

## Abstract

**Background:**

Mixed models are commonly used for the estimation of variance components and genetic evaluation of livestock populations. Some evaluation models include two types of additive genetic effects, direct and maternal. Estimates of variance components obtained with models that account for maternal effects have been the subject of a long-standing controversy about strong negative estimates of the covariance between direct and maternal effects. Genomic imprinting is known to be in some cases statistically confounded with maternal effects. In this study, we analysed the consequences of ignoring paternally inherited effects on the partitioning of genetic variance.

**Results:**

We showed that the existence of paternal parent-of-origin effects can bias the estimation of variance components when maternal effects are included in the evaluation model. Specifically, we demonstrated that adding a constraint on the genetic parameters of a maternal model resulted in correlations between relatives that were the same as those obtained with a model that fits only paternally inherited effects for most pairs of individuals, as in livestock pedigrees. The main consequence is an upward bias in the estimates of the direct and maternal additive genetic variances and a downward bias in the direct-maternal genetic covariance. This was confirmed by a simulation study that investigated five scenarios, with the trait affected by (1) only additive genetic effects, (2) only paternally inherited effects, (3) additive genetic and paternally inherited effects, (4) direct and maternal additive genetic effects and (5) direct and maternal additive genetic plus paternally inherited effects. For each scenario, the existence of a paternally inherited effect not accounted for by the estimation model resulted in a partitioning of the genetic variance according to the predicted pattern. In addition, a model comparison test confirmed that direct and maternal additive models and paternally inherited models provided an equivalent fit.

**Conclusions:**

Ignoring paternally inherited effects in the maternal models for genetic evaluation can lead to a specific pattern of bias in variance component estimates, which may account for the unexpectedly strong negative direct-maternal genetic correlations that are typically reported in the literature.

**Electronic supplementary material:**

The online version of this article (doi:10.1186/s12711-015-0141-5) contains supplementary material, which is available to authorized users.

## Background

Genetic evaluation based on mixed models [1] has become the main tool for prediction of breeding values in livestock populations. The most commonly used parameterization accounts for the direct polygenic additive genetic effect inherent to each individual and for several systematic effects (e.g., sex, herd, season, etc.), as well as a residual source of variation. However, some traits may also be affected by maternal genetic effects that refer to the influence from the genome of the dam (e.g., milk production of the dam). These maternal genetic effects are commonly considered in genetic evaluation models for some livestock populations [2, 3]. Nevertheless, variance components estimates that are obtained with models that account for maternal effects have been the subject of a long-standing controversy about the unexpectedly strong negative estimates of the covariance between direct and maternal genetic effects [4–6].

Genomic imprinting [7, 8] is the total or partial silencing of paternal or maternal alleles in the progeny. Several specific genes with imprinted inheritance have been identified in livestock species [9], such as the callipyge mutation in sheep [10, 11] and the *insulin*-*like growth factor 2* (*IGF2*) gene in pig [12, 13]. Imumorin et al. [14] also reported that genomic imprinting is involved in the determinism of quantitative trait loci (QTL) in cattle. More generally, genomic imprinting has been described as a widespread phenomenon in other mammals [15]. Studies on pig growth [16] and bovine fatness traits [17] have also provided statistical evidence for the existence of genetic variation associated with paternal parent-of-origin inheritance that can account for over 10 % of the phenotypic variability in these traits. More recently, Neugenbauer et al. [18, 19] and Tier and Meyer [20] confirmed this result on other cattle and pig traits.

Maternal effects can be statistically confounded with maternal inheritance, as noted by Hager et al. [21]. Furthermore, Meyer and Tier [22] suggested that ignoring parent-of-origin effects may be one of the possible causes for the unexpectedly large negative estimates of the correlation between direct and maternal genetic effects. In this paper, we provide further evidence that supports this latter argument by showing how the existence of a paternal parent-of-origin inheritance caused by the silencing of the alleles in the maternal gametes by imprinting can bias estimates of variance components in models that account for maternal effects. Specifically, we show that for any two individuals that were not related within the paternal lineage, direct and maternal additive genetic effects may generate the same expected covariance between relatives as paternal parent-of-origin or gametic effects. For most livestock pedigrees, the consequence of this is a downward bias in the estimate of the direct-maternal genetic covariance when paternal inheritance occurs and is not properly accounted for. To confirm this postulate, we developed a simulation experiment. In addition, we analysed a dataset of weight records at 210 days for the Pirenaica beef cattle population and we discuss the results in the light of this new evidence.

## Methods

### Quantitative genetic models

Consider a model with direct and maternal additive genetic effects. Hereafter, we use the subscripts “*o*” and “*m*” to differentiate between direct and maternal components, respectively. In addition, capital letters are used to identify different individuals. Under this model, the phenotypic values of individuals *A* and *B* are defined as:

$$ \begin{array}{c}\hfill {y}_A=\mu +{a}_{oA}+{a}_{m{A}_D}+{e}_A\hfill \\ {}\hfill {y}_B=\mu +{a}_{oB}+{a}_{m{B}_D}+{e}_B\hfill \end{array}, $$

where *μ* is the general mean, *a*_*oX*_ and *a*_*mX*_ are the direct and maternal additive genetic effects, respectively, and *e*_*X*_ is the residual for individual *X* = {*A*, *B*, *A*_*D*_, *B*_*D*_}. According to this notation, *A*_*D*_ and *B*_*D*_ stand for the dams of *A* and *B*, respectively.

Information for the estimation of variance components estimation originates from covariances between relatives. Under the model with maternal effects described above, and assuming independence of the residuals, the covariance between the phenotypic values of individuals *A* and *B* is:

$$ \begin{array}{l}\mathrm{c}\mathrm{o}\mathrm{v}\left({\mathrm{y}}_{\mathrm{A}},{\mathrm{y}}_{\mathrm{b}}\right)=\hfill \\ {}\mathrm{c}\mathrm{o}\mathrm{v}\left({\mathrm{a}}_{\mathrm{oA}},{\mathrm{a}}_{\mathrm{oB}}\right)+\mathrm{c}\mathrm{o}\mathrm{v}\left({\mathrm{a}}_{\mathrm{oA}},{{\mathrm{a}}_{\mathrm{m}\mathrm{B}}}_{{}_{\mathrm{D}}}\right)+\mathrm{c}\mathrm{o}\mathrm{v}\left({\mathrm{a}}_{\mathrm{m}{\mathrm{A}}_{\mathrm{D}}},{\mathrm{a}}_{\mathrm{oB}}\right)+\mathrm{c}\mathrm{o}\mathrm{v}\left({{\mathrm{a}}_{\mathrm{m}\mathrm{A}}}_{{}_{\mathrm{D}}},{{\mathrm{a}}_{\mathrm{m}\mathrm{B}}}_{{}_{\mathrm{D}}}\right)=\hfill \\ {}\mathrm{r}\left(\mathrm{A},\mathrm{B}\right){\sigma}_{\mathrm{a}}^2+\mathrm{r}\left(\mathrm{A},\mathrm{B}\right){\sigma}_{\mathrm{a}\mathrm{m}}+\mathrm{r}\left({\mathrm{A}}_{\mathrm{D}},\mathrm{B}\right){\sigma}_{\mathrm{a}\mathrm{m}}+\mathrm{r}\left({\mathrm{A}}_{\mathrm{D}},{\mathrm{B}}_{\mathrm{D}}\right){\sigma}_{\mathrm{m}}^2,\hfill \end{array} $$

where *σ*_a_^2^, *σ*_*m*_^2^, and *σ*_*am*_ are the direct additive genetic variance, the maternal additive genetic variance, and the direct-maternal genetic covariance, respectively, and *r*(*X*,*Y*) represents the additive relationship between individuals *X* and *Y*. In this context, the main sources of information to estimate maternal parameters are the observed correlations between phenotypes of full sibs (FS), maternal half sibs (MHS) and dam-offspring (DO) pairs. Equated to their expectations under the maternal model, the following expressions are obtained:

$$ \begin{array}{l}\operatorname{cov}\left(\mathrm{F}\mathrm{S}\right)=\frac{1}{2}{\sigma}_{\mathrm{a}}^2+{\sigma}_{\mathrm{a}\mathrm{m}}+{\sigma}_{\mathrm{m}}^2,\hfill \\ {}\operatorname{cov}\left(\mathrm{M}\mathrm{H}\mathrm{S}\right)=\frac{1}{4}{\sigma}_{\mathrm{a}}^2+{\sigma}_{\mathrm{a}\mathrm{m}}+{\sigma}_{\mathrm{m}}^2,\hfill \\ {}\operatorname{cov}\left(\mathrm{DO}\right)=\frac{1}{2}{\sigma}_{\mathrm{a}}^2+\frac{5}{4}{\sigma}_{\mathrm{a}\mathrm{m}}+\frac{1}{2}{\sigma}_{\mathrm{m}}^2.\hfill \end{array} $$

Now, let:

1 $$ {\sigma}_a^2=4{\sigma}_m^2=-2{\sigma}_{am}. $$

A little algebra shows that under condition (1) the previous expressions reduce to:

$$ \begin{array}{c}\hfill \operatorname{cov}\left(\mathrm{F}\mathrm{S}\right)=\frac{1}{4}{\sigma}_{\mathrm{a}}^2,\hfill \\ {}\hfill \operatorname{cov}\left(\mathrm{M}\mathrm{H}\mathrm{S}\right)=0,\hfill \\ {}\hfill \operatorname{cov}\left(\mathrm{DO}\right)=0.\hfill \end{array} $$

This is exactly the expectation of the covariance between relatives for these same relationships under a paternal gametic model of inheritance. In the Appendix section, we show that this result can be generalised to any genealogical relationship between a pair of individuals, on the condition that one was not an ancestor of the other within the paternal lineage. Specifically, the covariance between the phenotypic values of any two individuals (say *A* and *B*) under the condition set by Equation (1) is:

$$ \operatorname{cov}\left({\mathrm{y}}_{\mathrm{A}},{\mathrm{y}}_{\mathrm{B}}\right)=\frac{1}{4}\mathrm{r}\left({\mathrm{A}}_{\mathrm{S}},{\mathrm{B}}_{\mathrm{s}}\right){\sigma}_{\mathrm{a}}^2, $$

where *A*_*S*_ and *B*_*S*_ are the sires of *A* and *B*, respectively.

This striking result implies that the parameterization of the genetic covariance under the standard maternal animal model is confounded with a model that explains the whole genetic variation as paternally inherited; i.e.

$$ \mathrm{c}\mathrm{o}\mathrm{v}\left({\mathrm{y}}_{\mathrm{A}},{\mathrm{y}}_{\mathrm{B}}\right)={\mathrm{r}}_{\mathrm{g}}\left({\mathrm{A}}^{\mathrm{P}},{\mathrm{B}}^{\mathrm{P}}\right){\sigma}_{\mathrm{s}}^2, $$

where *r*_*g*_(*A*^*p*^,*B*^*p*^) is the gametic relationship [23] between paternal gametes of individuals *A* and *B* (*A*^*p*^ and *B*^*p*^) and *σ*_*s*_^2^ is the variance caused by paternally inherited gametic effects. Stated the other way around, the existence of paternally inherited variation that is not accounted for by the maternal model will tend to be assigned to the remaining variance components according to Equation (1) in order to accommodate the observed correlations between relatives. As a consequence, variance component estimation will explicitly inflate estimates of the direct and maternal additive genetic variances, with a magnitude close to twice and one half the paternally inherited variance, respectively, and it will reduce the direct-maternal genetic covariance by a value equal to the paternally inherited variance. The following section describes a simulation experiment that we developed in order to test this result.

### Simulation study

Each simulated dataset consisted of a base population of 1000 individuals (500 males and 500 females) and two generations of 5000 phenotyped individuals that comprised 2500 males and 2500 females. Phenotypic records were generated for the individuals of the last two generations based on the following model:

$$ \mathbf{y}=\mu +{\mathbf{Z}}_{\mathbf{a}}{\mathbf{a}}_{\mathbf{o}}+{\mathbf{Z}}_{\mathbf{m}}{\mathbf{a}}_{\mathbf{m}}+{\mathbf{Z}}_{\mathbf{s}}\mathbf{s}+\mathbf{e}, $$

where **y** is the vector of phenotypic data, *μ* is the general mean, set to 100 units, **a**_**o**_ and **a**_**m**_ are vectors of the direct and maternal genetic effects, **s** is a vector of paternally inherited gametic effects, and **e** is the vector of residuals, while **Z**_**a**_, **Z**_**m**_ and **Z**_**s**_ are incidence matrices. Covariances between genetic (**a**_**o**_ and **a**_**m**_) and gametic effects (**s**) were assumed to be zero and

$$ Var\left(\mathbf{e}\right)=\mathbf{I}{\sigma}_{\mathbf{e}}^2, $$

where **I** is the identity matrix and *σ*_**e**_^2^ is the residual variance. The variances of the direct and maternal additive genetic effects are:

$$ Var\left(\begin{array}{c}\hfill {\mathbf{a}}_{\mathbf{o}}\hfill \\ {}\hfill {\mathbf{a}}_{\mathbf{m}}\hfill \end{array}\right)=\mathbf{A}\otimes \mathbf{T}, $$

where **A** is the numerator relationship matrix and

$$ \mathbf{T}=\left(\begin{array}{cc}\hfill {\sigma}_a^2\hfill & \hfill {\sigma}_{am}\hfill \\ {}\hfill {\sigma}_{am}\hfill & \hfill {\sigma}_{{}_m}^2\hfill \end{array}\right), $$

For the paternally inherited gametic effects (**s**):

$$ Var\left(\mathbf{s}\right)=\mathbf{G}{\sigma}_s^2, $$

where **G** is the gametic relationship matrix

Five simulation scenarios were developed:

Scenario 1 corresponds to a pure direct additive model of inheritance with the following simulated parameters:

$$ {\sigma}_{\mathrm{a}}^2=500,{\sigma}_{\mathrm{m}}^2=0,{\sigma}_{am}=0,{\sigma}_{\mathrm{s}}^2=0,{\sigma}_{\mathrm{e}}^2=1000. $$

Scenario 2 assumes that the only source of genetic variation is the paternally inherited variance:

$$ {\sigma}_{\mathrm{a}}^2=0,{\sigma}_{\mathrm{m}}^2=0,{\sigma}_{am}=0,{\sigma}_{\mathrm{s}}^2=250,{\sigma}_{\mathrm{e}}^2=1000. $$

Scenario 3 combines both sources of genetic variation, direct additive and paternally inherited variance. The simulated parameters were:

$$ {\sigma}_{\mathrm{a}}^2=500,{\sigma}_{\mathrm{m}}^2=0,{\sigma}_{am}=0,{\sigma}_{\mathrm{s}}^2=250,{\sigma}_{\mathrm{e}}^2=1000. $$

Scenario 4 corresponds to the covariance structure that is assumed in a standard maternal animal model. A strong negative covariance between direct and maternal additive genetic effects was also simulated in order to mimic estimates that are frequently achieved in livestock populations for maternally influenced traits:

$$ {\sigma}_{\mathrm{a}}^2=500,{\sigma}_{\mathrm{m}}^2=250,{\sigma}_{am}=-250,{\sigma}_{\mathrm{s}}^2=0,{\sigma}_{\mathrm{e}}^2=1000. $$

Finally, Scenario 5 includes all three types of genetic effects: direct and maternal additive and paternally inherited. The simulated parameters were:

$$ {\sigma}_{\mathrm{a}}^2=500,{\sigma}_{\mathrm{m}}^2=250,{\sigma}_{am}=-250,{\sigma}_{\mathrm{s}}^2=250,{\sigma}_{\mathrm{e}}^2=1000. $$

For each of these simulation scenarios, a total of 10 independent populations were generated.

### Pirenaica beef cattle data

In addition to the simulated scenarios, we analysed data for phenotypic records on the Pirenaica beef cattle breed. The Pirenaica breed is a meat-type beef population from northern Spain with an approximate census of 20 000 individuals that are typically reared under extensive conditions [24]. The dataset consisted of 17 069 records for weight at 210 days (W210), with an average value of 266 kg and a raw standard deviation of 52.9 kg. Phenotypic records were adjusted according to the recommendations of the Beef Improvement Federation [25]. In addition, a pedigree file including 125 974 individual-sire-dam records was used. This information was provided by the National Breeders Confederation (*Confederación Nacional de Asociaciones de Ganado Pirenaico*-*CONASPI*; http://www.conaspi.es). Ethical approval for animal care and use was not required for this study since all data was field data from the Yield Recording System of the Pirenaica breed; furthermore, data was recorded by the stockbreeders themselves, under standard farm management, with no additional requirements.

An additional simulation study was performed based on the same scenarios as described in the previous section, but using genealogical information of the Pirenaica beef cattle breed and replacing each phenotypic record by a simulated value. The aim was to replicate the same structure of relationships between individuals of the real dataset.

### Statistical models of estimation

Although a realistic implementation of all potential genetic sources of variation in the same hierarchical mixed linear model may result in non-estimable functions, the description of the full model is essential to understand the simplified parameterizations that are used below. The full statistical model is:

$$ \mathbf{y}=\mathbf{X}\mathbf{b}+{\mathbf{Z}}_{\mathbf{a}}{\mathbf{a}}_{\mathbf{o}}+{\mathbf{Z}}_{\mathbf{m}}{\mathbf{a}}_{\mathbf{m}}+{\mathbf{Z}}_{\mathbf{s}}\mathbf{s}+{\mathbf{Z}}_{\mathbf{d}}\mathbf{d}+{\mathbf{Z}}_{\mathbf{p}}\mathbf{p}+{\mathbf{Z}}_{\mathbf{h}}\mathbf{h}+\mathbf{e}, $$

where **y** is the vector of phenotypic data, **b** is the vector of systematic effects, consisting of the general mean for the simulated dataset and the two sexes and 16 age-groups of dam age of parity for the Pirenaica beef cattle data, and **a**_**o**_, **a**_**m**_, **s** and **d** are vectors of direct additive genetic, maternal additive genetic, paternal gametic and maternal gametic effects, respectively. Finally, **p** and **h** are vectors of random permanent maternal (9224 levels) and herd-year-season (2781 levels) environmental effects (not included in the simulated datasets), **e** is a vector of residuals, and **X**, **Z**_**a**_, **Z**_**m**_, **Z**_**s**_, **Z**_**d**_, **Z**_**p**_, and **Z**_**h**_ are incidence matrices that link the effects with the phenotypic data. Variances and covariances for random sources of variation were defined as follows:

$$ \operatorname{var}\left[\begin{array}{l}{\mathbf{a}}_o\hfill \\ {}{\mathbf{a}}_m\hfill \\ {}\mathbf{s}\hfill \\ {}\mathbf{d}\hfill \\ {}\mathbf{p}\hfill \\ {}\mathbf{h}\hfill \\ {}\mathbf{e}\hfill \end{array}\right]=\left[\begin{array}{lllllll}\mathbf{A}{\sigma}_a^2\hfill & \mathbf{A}{\sigma}_{am}\hfill & 0\hfill & 0\hfill & 0\hfill & 0\hfill & 0\hfill \\ {}\mathbf{A}{\sigma}_{am}\hfill & \mathbf{A}{\sigma}_m^2\hfill & 0\hfill & 0\hfill & 0\hfill & 0\hfill & 0\hfill \\ {}0\hfill & 0\hfill & \mathbf{G}{\sigma}_s^2\hfill & \mathbf{G}{\sigma}_{sd}\hfill & 0\hfill & 0\hfill & 0\hfill \\ {}0\hfill & 0\hfill & \mathbf{G}{\sigma}_{sd}\hfill & \mathbf{G}{\sigma}_d^2\hfill & 0\hfill & 0\hfill & 0\hfill \\ {}0\hfill & 0\hfill & 0\hfill & 0\hfill & \mathrm{I}{\sigma}_p^2\hfill & 0\hfill & 0\hfill \\ {}0\hfill & 0\hfill & 0\hfill & 0\hfill & 0\hfill & \mathbf{I}{\sigma}_h^2\hfill & 0\hfill \\ {}0\hfill & 0\hfill & 0\hfill & 0\hfill & 0\hfill & 0\hfill & \mathbf{I}{\sigma}_e^2\hfill \end{array}\right], $$

where *σ*_*p*_^2^ and *σ*_*h*_^2^ represent the variances of the permanent maternal and herd-year-season effects, *σ*_*d*_^2^ is the variance of maternal gametic effects, and *σ*_*sd*_ is the covariance between paternal and maternal gametic effects. All other parameters were defined previously. Note that under a pure direct additive model:

2 $$ {\sigma}_{\mathrm{s}}^2={\sigma}_{\mathrm{d}}^2={\sigma}_{\mathrm{s}\mathrm{d}}=\frac{1}{2}{\sigma}_{\mathrm{a}}^2. $$

A number of models based on reduced parameterizations of this full model were fitted to both the simulated and the real data:

- Model A: **y** **=** **Xb** **+** **Z**_**a**_**a**_**o**_ **+** **e,**
- Model S: **y** **=** **Xb** **+** **Z**_***s***_**s** **+** **e,**
- Model AS: **y** **=** **Xb** **+** **Z**_**a**_**a**_**o**_ **+** **Z**_***s***_**s** **+** **e,**
- Model SD: **y** **=** **Xb** **+** **Z**_**s**_**s** **+** **Z**_**d**_**d** + **e,**
- Model AM: **y** **=** **Xb** **+** **Z**_**a**_**a**_**o**_ **+** **Z**_**m**_**a**_**m**_ **+** **e,**
- Model AMS: **y** **=** **Xb** **+** **Z**_**a**_**a**_**o**_ **+** **Z**_**m**_**a**_**m**_ **+** **Z**_**s**_**s** **+** **e,**

When the Pirenaica beef data was used, all models also included the terms **z**_**p**_**p** and **z**_**h**_**h**.

For statistical analysis, we applied a Bayesian approach through a Gibbs sampler [26]. The prior distributions for systematic effects and variance components were assumed to be bounded uniform, and the prior distributions for the genetic effects were multivariate Gaussian distributions with mean zero and variance as defined in the previous section. Analyses were run for each simulated or real dataset as a single chain of 525 000 cycles with the first 25 000 iterations being discarded. Convergence was checked by visual inspection of the chains and by applying the test of Raftery and Lewis [27]. All samples were stored to calculate summary statistics.

### Model comparison

Models were compared using the pseudo log-marginal probability of the phenotypic data [28]. If we consider the data vector **y** = (*y*_i,_**y**_-*i*_), where *y*_i_ is the *i*^th^ datum and **y**_-i_ is the vector of data with *i*^th^ datum deleted, the conditional predictive distribution has a probability density equal to:

$$ \mathrm{p}\left({\mathrm{y}}_i\Big|{\mathbf{y}}_{-\mathrm{i}}\right)={\displaystyle \int \mathrm{p}\left({\mathrm{y}}_i\Big|{\mathbf{y}}_{-\mathrm{i}}\right)\mathrm{f}}\left(\boldsymbol{\uptheta} \Big|{\mathbf{y}}_{-\mathrm{i}}\right)\mathrm{d}\mathbf{y}, $$

where **θ** is the vector of parameters. Therefore, *p*(*y*_*i*_|**y**_-*i*_) can be interpreted as the probability of each datum given the rest of the data and is known as the conditional predictive ordinate (CPO) for the *i*^th^ datum. The pseudo log-marginal probability of the data (LogCPO) is then:

$$ {\displaystyle \sum_i \ln p\left({y}_i\Big|{\mathbf{y}}_{-i}\right)}. $$

The collection of conditional predictive densities is equivalent to the marginal probability of the data [29]. A Monte Carlo approximation of the LogCPO suggested by Gelfand [28] is:

$$ {\displaystyle \sum_i \ln \widehat{p}\left({y}_i\Big|{\mathbf{y}}_{-i}\right)}, $$

$$ \mathrm{where}\kern2em \widehat{p}\left({y}_i\Big|{\mathbf{y}}_{-i}\right)=N{\left[{\displaystyle \sum_{j=1}^N\frac{1}{p\left({y}_i\Big|{\theta}^j\right)}}\right]}^{-1}, $$

and *N* is the number of Markov chain Monte Carlo (McMC) draws, and *θ*^*j*^ is the *j*^th^ draw from the posterior distribution of the corresponding parameter. The interpretation of the results of this test is that the larger is LogCPO, the higher is the marginal probability of data and, thus, the better is the relative fit.

## Results

### Scenario 1: additive model of inheritance

In Scenario 1, all genetic variation was generated as direct additive; there were no maternal genetic or paternally inherited effects. The average posterior mean estimates of variance components and ratios of components of variance are in Table 1. When Model A was used, the estimate of the direct additive genetic variance almost matched the simulated value. Furthermore, when the estimation model only included paternal gametic effects (Model S), a significant proportion of the variation was assigned to these effects and the remaining additive variation was incorporated into the residual variance. Implementation of a more complex parameterization, such as Model AS, attributed a small, although negligible, amount of the simulated variability to the paternally inherited effects. As expected, Model SD absorbed close to half of the additive genetic variance of the paternal gametic effects and another half of the maternal effects. In addition, the correlation between paternal and maternal effects was close to 1. Finally, the models that included an additive maternal genetic effect (AM and AMS) also correctly assigned most genetic variation to the direct additive effects, and only small amounts to other variance components. The results of the model comparison test showed that Models A and SD presented the best fit, followed closely by Models AM, AS and AMS. Model S had the worst fit.

**Table 1:** Averages of posterior means (and standard deviations) of (co)variance components for Scenario 1 (*σ*_*a*_^2^ = 500, *σ*_*e*_^2^ = 1000)

Parameter	Model of estimation					
	A	S	AS	SD	AM	AMS
*σ*_*a*_^2^	514.8 (33.2)	-	498.7 (34.8)	-	512.5 (43.0)	469.6 (51.5)
*σ*_*m*_^2^	-	-	-	-	28.6 (9.6)	25.5 (8.9)
*σ*_*am*_	-	-	-	-	−14.9 (18.65)	0.88 (19.5)
*σ*_*s*_^2^	-	292.4 (27.5)	27.4 (16.3)	239.7 (24.2)	-	30.4 (19.2)
*σ*_*d*_^2^	-	-	-	284.7 (25.2)	-	.
*σ*_*sd*_	-	-	-	239.8 (21.9)	-	-
*σ*_*e*_^2^	1010.8 (26.4)	1212.8 (26.5)	1001.8 (26.8)	1001.3 (29.89)	1003.7 (29.0)	1005.8 (29.0)
_L_	−50209.08	−50517.54	−50210.99	−50208.96	−50213.5	−50215.8

*σ*_*a*_^2^ and *σ*_*m*_^2^ are the direct and maternal additive genetic variances and *σ*_*am*_ is the direct-maternal genetic covariance. *σ*_*s*_^2^ and *σ*_*d*_^2^ are the paternal and maternal gametic variances and *σ*_*sd*_ is the covariance between the paternal and maternal gametic effects. Finally, *σ*_*e*_^2^ is the residual variance and L is the average logCPO across 10 replicates.

### Scenario 2: paternal inheritance model

Scenario 2 (Table 2) only included a variance of paternally inherited effects of 250, without considering any other source of genetic variance. The average posterior mean estimate of variance due to paternally inherited effects under Model S was close to the simulated value. However, with Model A, estimates of direct additive genetic variance were on average close to 150, although this variance component was not included in the simulation process. Model AS assigned most of the variance to paternally inherited effects and a small amount to the direct additive effect. Model SD assigned most of the genetic variance to paternal gametic effects, although negligible amounts of variability were also assigned to the gametic maternal variance component. Model AM, which corresponds to the standard maternal animal model, strongly overestimated the direct and maternal additive genetic variances, and resulted in a negative estimate of the covariance between direct and maternal additive genetic effects, which resulted in a negative correlation close to −1. This tendency was reduced when paternally inherited effects were included in the model (Model AMS). Average posterior mean estimates of variances of both direct and maternal additive genetic effects and the covariance between them were smaller than in Model AM. However, the genetic correlation was still negative (−0.90) and a substantial percentage of variability was assigned to paternally inherited effects. It should be noted that the residual variance was inflated in Model A and smaller than the simulated value in Model AM. The model with the best fit was Model S, which mimicked the simulation model, and was closely followed by Models AS and SD, which included paternally inherited effects, and by Model AM. Model A had the worst fit.

**Table 2:** Averages of posterior means (and posterior standard deviations) of (co)variance components for Scenario 2 (*σ*_*s*_^2^ = 250, *σ*_*e*_^2^ = 1000)

Parameter	Model of estimation					
	A	S	AS	SD	AM	AMS
*σ*_*a*_^2^	147.5(18.7)	-	21.3(13.6)	-	372.2 (43.8)	111.4 (50.7)
*σ*_*m*_^2^	-	-	-	-	105.7 (21.8)	40.8 (19.0)
*σ*_*am*_	-	-	-	-	−191.9 (16.6)	−60.9 (29.8)
*σ*_*s*_^2^	-	242.0 (31.5)	208.1(22.9)	218.6(16.6)	-	162.0 (30.2)
*σ*_*d*_^2^	-	-	-	14.6(9.8)	-	-
*σ*_*sd*_	-	-	-	1.7(16.6)	-	-
*σ*_*e*_^2^	1101.6(21.9)	990.3(31.1)	1019.7(23.1)	1016.1(23.6)	960.8 (30.1)	997.3 (26.9)
L	−49730.0	−49609.5	−49612.4	−49614.0	−49613.2	−49613.3

*σ*_*a*_^2^ and *σ*_*m*_^2^ are the direct and maternal additive genetic variances and *σ*_*am*_ is the direct-maternal genetic covariance. *σ*_*s*_^2^ and *σ*_*d*_^2^ are the paternal and maternal gametic variances and *σ*_*sd*_ is the covariance between the paternal and maternal gametic effects. Finally, *σ*_*e*_^2^ is the residual variance and L is the average logCPO across 10 replicates.

### Scenario 3: additive and paternal inheritance model

Scenario 3 included an additive genetic variance component of 500 and a paternally inherited component with variance equal to 250. The results are in Table 3. The first and second models of estimation (Models A and S) clearly overestimated the additive genetic variance and the paternal gametic variance, respectively. This overestimation disappeared with Model AS, which replicated the model of simulation by including a paternal gametic effect, and the estimates were close to the simulated values. Models AM and AMS overestimated the additive genetic variance and assigned some of the variance to the maternal genetic effects. Furthermore, these models generated a negative covariance between additive and maternal genetic effects. This overestimation of variances was reduced with Model AMS, for which a substantial amount of the variability was assigned to the paternally inherited effects, although less than the simulated value. The model comparison test indicated that Models AS, SD, AM and AMS had the best fit, with similar average LogCPO values, whereas Models A and S had a substantially poorer fit.

**Table 3:** Averages of posterior means (and posterior standard deviations) of (co)variance components for Scenario 3 (*σ*_*a*_^2^ = 500, *σ*_*s*_^2^ = 250,  *σ*_*e*_^2^ = 1000)

Parameter	Model of estimation					
	A	S	AS	SD	AM	AMS
*σ*_*a*_^2^	672.1 (37.6)	-	536.7 (39.8)	-	872.0 (60.0)	692.4 (97.8)
*σ*_*m*_^2^	-	-	-	-	96.1 (22.8)	57.7 (21.1)
*σ*_*am*_	-	-	-	-	−181.1 (35.7)	−94.5 (44.6)
*σ*_*s*_^2^	-	566.3 (40.5)	201.4 (38.0)	475.5 (35.7)	-	116.8 (60.4)
*σ*_*d*_^2^	-	-	-	256.0 (26.2)	-	-
*σ*_*sd*_	-	-	-	281.3 (30.8)	-	-
*σ*_*e*_^2^	1091.0 (28.8)	1185.7 (37.0)	1025.8 (32.6)	1034.5 (35.1)	972.9 (37.0)	992.4 (35.4)
_L_	−50857.1	−51040.3	−50811.7	−50810.5	−50813.5	−50812.9

*σ*_*a*_^2^ and *σ*_*m*_^2^ are the direct and maternal additive genetic variances and *σ*_*am*_ is the direct-maternal genetic covariance. *σ*_*s*_^2^ and *σ*_*d*_^2^ are the paternal and maternal gametic variances and *σ*_*sd*_ is the covariance between the paternal and maternal gametic effects. Finally, *σ*_*e*_^2^ is the residual variance and L is the average logCPO across10 replicates.

### Scenario 4: maternal animal model

Scenario 4 corresponds to the standard maternal animal model. Direct and maternal additive genetic variances of 500 and 250 were generated, but with a strong negative correlation between these two effects (−0.707). Average posterior mean estimates are in Table 4. As expected, the average posterior mean estimates from Model AM almost matched the simulated values for the direct and maternal additive genetic effects, and the covariance between them. Moreover, all models except AM gave biased estimates of variance components. In particular, Model A resulted in an average posterior mean estimate of the direct additive genetic variance that was smaller than the simulated value. Model S assigned a significant proportion of the variation to paternally inherited effects, although they were not included in the simulation model. This was confirmed with Model AS, which assigned a significant proportion of the variation to the paternally inherited effects. Model SD assigned a significant proportion of the variance to both paternal and maternal gametic effects, but the covariance between them was almost null. Finally, with Model AMS, the presence of a significant amount of variation in the paternal gametic effects reduced the magnitude of the direct and maternal additive genetic variance components compared to the results obtained with Model AM. For this scenario, the models with the best fit were SD and AM, followed by Models AS and S. The models that fitted the simulated data worst were A and AMS.

**Table 4:** Averages of posterior means (and posterior standard deviations) of (co)variance components for Scenario 4 (*σ*_*a*_^2^ = 500, *σ*_*m*_^2^ = 250,  *σ*_*am*_ = − 250, *σ*_*e*_^2^ = 1000)

Parameter	Model of estimation					
	A	S	AS	SD	AM	AMS
*σ*_*a*_^2^	348.0 (28.5)	-	264.0 (24.2)	-	523.4 (47.0)	367.5 (76.5)
*σ*_*m*_^2^	-	-	-	-	268.8 (30.2)	228.8 (38.2)
*σ*_*am*_	-	-	-	-	−266.3 (33.1)	−187.1 (49.4)
*σ*_*s*_^2^	-	282.6 (25.7)	173.3 (29.2)	284.5 (29.8)	-	92.9 (41.3)
*σ*_*d*_^2^	-	-	-	237.4 (25.3)	-	-
*σ*_*sd*_	-	-	-	0.3 (25.3)	-	-
*σ*_*e*_^2^	1159.0 (21.1)	1224.6 (24.4)	1077.7 (25.3)	988.6 (24.9)	986.1 (24.6)	1012.0 (23.4)
L	−50427.0	−50375.2	−50329.7	−50318.3	−50318.7	−50494.3

*σ*_*a*_^2^ and *σ*_*m*_^2^ are the direct and maternal additive genetic variances and *σ*_*am*_ is the direct-maternal genetic covariance. *σ*_*s*_^2^ and *σ*_*d*_^2^ are the paternal and maternal gametic variances and *σ*_*sd*_ is the covariance between the paternal and maternal gametic effects. Finally, *σ*_*e*_^2^ is the residual variance and L is the average logCPO across 10 replicates.

### Scenario 5: direct, maternal and paternal inheritance model

Scenario 5 included direct and maternal additive genetic, and paternal gametic effects. Direct and maternal additive genetic effects with variances of 500 and 250 were generated, with a strong negative correlation between them (−0.707). The paternally inherited variance was 250. The average posterior mean estimates are in Table 5. The average results of Model AMS were very close to the simulated values. However, the standard deviation of the estimates was substantially higher than for the other models and also than for the previous scenarios. The results of Model A were very close to the simulated value for the direct additive genetic effect (510.8 vs 500), although residual variance was significantly overestimated. Model S, AS and SD overestimated the paternal gametic variance, which captured the sources of variation that were ignored in the estimation model. It is remarkable that Model SD provided almost a null covariance between sire and dam gametic effects. Finally, the results of Model AM reflected a redistribution of the ignored paternally inherited variance by increasing the direct and maternal additive genetic variances (to 924.6 and 371.0, respectively), and by generating a strong negative direct-maternal genetic covariance (−473.5). The results of the LogCPO test indicated that Model AMS, which replicated the simulation model, had the best fit. It was closely followed by Models AM and SD, whereas Models AS, S and A fitted the simulated data worst.

**Table 5:** Averages of posterior means (and posterior standard deviations) of (co)variance components for Scenario 5 (*σ*_*a*_^2^ = 500, *σ*_*m*_^2^ = 250,  *σ*_*am*_ = − 250, *σ*_*s*_^2^ = 250, *σ*_*e*_^2^ = 1000)

Parameter	Model of estimation					
	A	S	AS	SD	AM	AMS
*σ*_*a*_^2^	510.82 (30.39)	-	290.21 (24.10)	-	924.60 (50.17)	569.54 (121.68)
*σ*_*m*_^2^	-	-	-	-	370.97 (26.72)	279.33 (34.50)
*σ*_*am*_	-	-	-	-	−473.53 (29.09)	−292.76 (58.38)
*σ*_*s*_^2^	-	551.14 (37.39)	414.96 (36.31)	544.03 (35.98)	-	223.57 (77.09)
*σ*_*d*_^2^	-	-	-	236.24 (19.56)	-	-
*σ*_*sd*_	-	-	-	−5.05 (28.14)	-	-
*σ*_*e*_^2^	1236.37 (24.73)	1207.52 (29.87)	1056.10 (31.02)	973.73 (33.36)	923.52 (32.99)	973.03 (32.86)
L	−51048.4	−51010.0	−50902.3	−50868.6	−50860.6	−50858.1

*σ*_*a*_^2^ and *σ*_*m*_^2^ are the direct and maternal additive genetic variances and *σ*_*am*_ is the direct-maternal genetic covariance. *σ*_*s*_^2^ and *σ*_*d*_^2^ are the paternal and maternal gametic variances and *σ*_*sd*_ is the covariance between the paternal and maternal gametic effects. Finally, *σ*_*e*_^2^ is the residual variance and L is the average logCPO across 10 replicates

### Pirenaica beef cattle breed data

The results of the analysis using real data on the Pirenaica beef cattle breed are in Table 6. With Model A, the posterior mean estimate of the direct heritability (h^2^_d_) was equal to 0.367, but with Model AS, it was only equal to 0.174, whereas the posterior mean estimate of paternal gametic heritability was 0.379, which increased to 0.505 with Model S that did not take direct additive genetic effects into account. Model SD returned the highest posterior mean estimate of the paternal heritability (0.463), a lower value for maternal heritability (0.130) and a very low estimate of the direct-maternal genetic correlation (0.059). The standard maternal animal model (AM) produced a very large posterior estimate of direct additive heritability (0.471), along with a smaller posterior estimate of maternal heritability (0.157), and the correlation between direct and maternal effects was strong and negative (−0.734). Finally, with Model AMS, both heritabilities fell to very low values (0.045 and 0.056) and a significant proportion of the genetic variation was assigned to the paternal gametic effects (0.436). The results of the comparison of models based on LogCPO showed that Model SD had the best predictive ability, followed by Models AM and ASs. Model AMS was next, and Models S and A showed the worst fit.

**Table 6:** Posterior means (and posterior standard deviations) of (co)variance components for weight at 210 days in the Pirenaica beef cattle population

Parameter	Model of estimation					
	A	S	AS	SD	AM	AMS
*σ*_*a*_^2^	798.3 (52.0)	-	418.8 (60.7)	-	1317.0 (93.2)	94.0 (35.9)
*σ*_*m*_^2^	-	-	-	-	426.7 (54.1)	139.3 (30.1)
*σ*_*am*_	-	-	-	-	−553.9 (64.3)	31.2 (26.5)
*σ*_*s*_^2^	-	1236.2 (93.4)	891.3 (100.5)	1100.0 (96.1)	-	1029.8 (90.0)
*σ*_*d*_^2^	-	-	-	326.3 (43.2)	-	.
*σ*_*sd*_	-	-	-	30.4 (53.4)	-	-
*σ*_*h*_^2^	534.4 (24.6)	477.6 (23.0)	454.4 (22.1)	446.5 (22.2)	485.7 (23.4)	443.2 (22.0)
*σ*_*p*_^2^	94.5 (17.2)	191.2 (17.2)	109.2 (18.3)	69.1 (20.1)	52.8 (22.9)	47.9 (22.9)
*σ*_*e*_^2^	747.7 (32.4)	539.4 (48.4)	501.9 (43.9)	445.5 (53.9)	492.3 (46.9)	591.1 (47.5)
*h*_*a*_^2^	0.367 (0.021)	-	0.176 (0.026)	-	0.474 (0.023)	0.040 (0.015)
*h*_*m*_^2^	-	-	-	-	0.153 (0.016)	0.059 (0.012)
*r*_*am*_	-	-	-	-	−0.739 (0.016)	0.311 (0.187)
*h*_*s*_^2^	-	0.505 (0.030)	0.375 (0.037)	0.460 (0.032)	-	0.439 (0.032)
*h*_*d*_^2^	-	-	-	0.137 (0.018)	-	-
*r*_*sd*_	-	-	-	0.053 (0.091)	-	-
*c*_*h*_^2^	0.246 (0.010)	0.195 (0.010)	0.191 (0.009)	0.187 (0.010)	0.175 (0.009)	0.189 (0.010)
*c*_*p*_^2^	0.043 (0.008)	0.078 (0.007)	0.046 (0.008)	0.029 (0.008)	0.019 (0.008)	0.020 (0.010)
_LogCPO_	−86767	−86703	−86513	−86178	−86378	−86647

*σ*_*a*_^2^ and *σ*_*m*_^2^ are the direct and maternal additive genetic variances and *σ*_*am*_ is the direct-maternal genetic covariance. *σ*_*s*_^2^ and *σ*_*d*_^2^ are the paternal and maternal gametic variances and *σ*_*sd*_ is the covariance between the paternal and maternal gametic effects. *σ*_*h*_^2^ and *σ*_*p*_^2^ are the herd and maternal permanent environmental variances. *σ*_*e*_^2^ is the residual variance. *h*_*a*_^2^, *h*_*m*_^2^, *h*_*s*_^2^ and *h*_*d*_^2^ are the ratios of the direct additive genetic, maternal additive genetic, paternal gametic and maternal gametic variances to the phenotypic variance, respectively. *r*_*am*_ is the genetic correlation between direct and maternal additive effects and *r*_*sd*_ is the correlation between paternal and maternal gametic effects. *c*_*h*_^2^ and *c*_*p*_^2^ are the ratios of herd and permanent maternal environmental effects to the phenotypic variance.

## Discussion

We present a theoretical development that shows that the structure of the (co) variance components assumed by the standard maternal animal model is partially confounded with a model that considers a paternal gametic effect. Meyer and Tier [22] postulated that this may be one possible cause for the strong negative estimates of the genetic correlation between direct and maternal genetic effects that are typically obtained. We derived results that show that, for most of the genetic relationships in livestock pedigrees, the covariance between relatives generated by a paternally inherited effect is mimicked by the maternal animal model when variance components follow the pattern defined by Equation (1). Thus, when paternally inherited effects are ignored in the estimation model, its variance is assigned to the remaining variance components in order to accommodate the observed correlations between relatives. Under the maternal animal model, its consequences are an inflation of the direct and maternal additive genetic variances, and a downward bias of the direct-maternal genetic correlation.

These results were confirmed by the simulation study. When simulation and estimation models were analogous, the Bayesian analysis was able to provide suitable estimates of the variance components. Furthermore, when the true simulation structure of the (co)variance components was included in the estimation model together with other variance components, the procedure correctly estimated the true parameters, whereas estimates of the non-simulated variance components were small. However, the presence of paternal gametic effects (Scenarios 2 and 3) and their absence in the estimation model resulted in an overestimation of the direct additive genetic variance, as previously described by other authors [17, 22]. Furthermore, when paternally inherited effects that play a significant role in the genetic variation were ignored in a model that included correlated direct and maternal additive genetic effects, the direct additive genetic variance component was increased by almost twice the paternal gametic variance, whereas the genetic covariance between direct and maternal effects became negative and the estimate of the maternal additive genetic variance increased by almost one half of the simulated paternal gametic variance, as was observed in Scenarios 2, 3 and 5. As described above, this redistribution of the gametic paternal variance is linked to the constraint imposed in Equation (1). These results were also consistent with the presence of a significant percentage of paternal gametic variance for Models S, AS, SD and AMS in Scenario 4, where only direct and maternal additive effects were simulated, and with the increase of paternal gametic variance in Models S, AS and SD in Scenario 5. Finally, the decrease in covariance between paternal and maternal effects under Model SD in Scenarios 4 and 5 may also be attributed to this phenomenon.

More generally, when any relevant variance component in the simulation model was not considered in the estimation, substantial biases on the variance component estimation were noted. For example, ignoring a true direct additive genetic effect while assuming paternal and maternal gametic effects in the estimation model, led to the absorption of close to half of the additive genetic variance by the paternal gametic variance and half by the maternal gametic variance. In addition, the increase in the estimate of the covariance between paternal and maternal gametic effects was also of this magnitude. This distribution of the direct additive variance followed the pattern described in Equation (2) and was exploited in the model proposed by Neugenbauer et al. [18, 19] in order to detect paternal and maternal inherited effects.

The model comparison test that we performed provided support to our main argument. To illustrate this point, in Scenarios 2 and 3, where paternally inherited effects were included in the simulation model, the goodness of fit of Model AM was very close to that obtained with Models S and AS, which mimicked the respective simulation models. Moreover, under Scenarios 4 and 5, Model SD performed almost as well as Models AM or AMS, which replicated the true simulation models. Clearly, the constraint imposed by Equation (1) explains the similarity observed between the maternal animal model and the paternally inherited model in those cases.

It should be emphasized that the constraint imposed by Equation (1) is not a pure parametric equivalence, since it is limited by the condition that available correlations between relatives do not involve genealogical relationships where one individual is an ancestor of the other within the paternal lineage. Therefore, the ability to separate direct and maternal additive genetic effects and sire imprinted effects can only be achieved if relevant records from sires and their offspring are available or, more generally, if there exist records from individuals that are genetically linked through the paternal pathway. However, in beef cattle datasets, phenotypes on sire-offspring pairs are usually rare. In the Pirenaica beef cattle dataset analyzed here, there were 527 463 paternal half-sibs, 15 128 maternal half-sibs, 2707 full-sibs, 2411 dam-progeny and only 4566 sire-progeny pairs with phenotypic information on both individuals. Thus, most of the information available for estimation of variance components was provided by half-sib relatives, whose covariance generated by paternally inherited effects can be also achieved by the maternal animal model under the constraint imposed by Equation (1).

This latter explanation helps to understand the results of the analysis of the Pirenaica beef cattle dataset. Estimates of variance components obtained from Model A were concordant with previous results obtained in the same [30] and other beef cattle populations [31, 32]. In turn, alternative estimation models, such as S, AS and SD, suggested that paternal gametic effects may play an important role in the variation of weight at 210 days in this breed. In contrast, under Model AM, maternal additive effects were also very relevant. Furthermore, when both effects were fitted in the estimation model (AMS), the maternal effects contributed less to the total variation than the paternal gametic effects. A similar reduction in the maternal contribution on weaning weight was also found in the Bruna dels Pirineus beef cattle breed when paternal gametic effects were considered in the estimation model [33]. Indeed, more evidence for the presence of parent-of-origin variation is derived from the fact that when Model SD was used, a very low correlation between paternal and maternal gametic effects was obtained. This is in contrast to the results reported by Neugenbauer *et al*. [18, 19], which provided a high correlation between them, as in the outcome of the first simulation scenario, where a pure additive model was simulated. However, as observed with Scenarios 4 and 5, independence between paternal and maternal gametic effects, together with the presence of a significant paternally inherited variance component can also be due to the existence of a strong and negative genetic correlation between direct and maternal genetic effects. Given the structure of the information in the Pirenaica dataset, the genetic variance is distributed between paternal gametic and direct and maternal effects depending on the model of estimation and, thus, it is very difficult to clearly identify the true sources of variation. However, at this point, it is worth mentioning that the results of the simulation study using the Pirenaica’s breed genealogical structure were similar to those obtained with the above described simulation study (results in Tables S1, S2, S3, S4 and S5 [See Additional file 1: Tables S1-S5]). This allowed us to rule out the possibility of a statistical artefact caused by the structure of the information. Nevertheless, the model comparison using logCPO indicated that Model SD was most suited to the Pirenaica beef cattle data. It should be noted that this model performed well in all simulation scenarios (Tables 1, 2, 3, 4 and 5), since it can accommodate a significant amount of paternal inheritance and also a strong negative correlation between direct or maternal effects, as in Scenario 4. In fact, Model AM had the second best predictive ability based on the LogCPO approach and showed a better fit than Model AS. However, the very large difference in LogCPO between Models SD and AM (−86178 vs −86378) and the results of Model AMS suggest that the existence of significant paternal gametic effects is plausible in the Pirenaica population.

Many studies have reported negative estimates of the genetic covariance between direct and maternal additive effects for beef cattle populations [4–6]. Several explanations have been proposed, including environmental correlations between progeny performance and maternal environmental effects [34, 35] and sire × herd interactions [36, 37]. An additional explanation that should be considered is the presence of paternally inherited effects, as previously suggested by Meyer and Tier [22]. Our evidence is consistent with the effect associated with imprinted genes, such as the *insulin*-*like growth factor 2* (*IGF2*) gene involved in fetal growth [38, 39] or postnatal growth and fat deposition in pigs [11, 12, 40] and mice [41]. Genomic imprinting of the *IGF2* gene has also been shown to affect meat quality traits in beef cattle [42, 43] and some mutations in *IGF2* have been associated with carcass traits [44] and body weight [45].

Furthermore, a wealth of evidence on the role of parental inheritance in fetal and early growth in mammals supports the imprinting hypothesis [46, 47]. This is related with parent-offspring conflict theories about the evolutionary origin of paternal effects [48], which are based on the hypothesis that the self-interest of the paternal genome is to increase the growth of the fetus or young individuals, whereas the aim of the maternal genome is to sufficiently limit growth to ensure the success of future offspring without jeopardizing the mother’s survival. According to this hypothesis, genes that enhance growth tend to be more expressed if they are paternally inherited than when they are maternally inherited. This phenomenon should be even more marked in species with a low rate or absence of full siblings and a high frequency of multiple paternity [49], as is the case for cattle.

Finally, other causes that can explain overestimation of paternal gametic variance are a large difference in allele frequencies between sires and dams or the presence of significant dominance variation. However, these are not relevant for close populations and that include few full-sib relationships, respectively. In future studies, a very promising alternative to distinguish between paternally and maternally inherited effects arises from the use of molecular information that may contribute to better discriminate between paternal and maternal alleles.

## Conclusions

Ignoring paternally inherited effects in genetic evaluations with maternal animal models can lead to a specific pattern of bias in the estimates of variance components. This phenomenon may account for the unexpectedly strong negative direct-maternal genetic correlations that are reported in the literature. In addition, we showed that the existence of paternal effects may play a role in the explanation of the partitioning of genetic variation in growth traits and possibly in some other relevant traits in livestock production. The main implication of this study is that the presence of relevant paternally inherited variation should be investigated when the maternal animal model provided negative correlation estimates between direct and maternal effects. In addition, in those cases, applying maternal animal models should be done with caution.

## Appendix

Consider a model that fits direct and maternal additive genetic effects. As pointed out in the Methods section, the phenotypic values of any two individuals, say *A* and *B*, are modelled as:

$$ \begin{array}{c}\hfill {y}_A=\mu +{a}_{oA}+{a_{mA}}_{{}_D}+{e}_A\hfill \\ {}\hfill {y}_B=\mu +{a}_{oB}+{a_{mB}}_{{}_D}+{e}_B\hfill \end{array}. $$

Assuming independence of the residuals, the covariance between both phenotypic values can be described as a function of the following four pairwise covariances:

$$ \begin{array}{c}\mathrm{c}\mathrm{o}\mathrm{v}\left({\mathrm{y}}_{\mathrm{A}},{\mathrm{y}}_{\mathrm{B}}\right)=\mathrm{c}\mathrm{o}\mathrm{v}\left({\mathrm{a}}_{\mathrm{oA}},{\mathrm{a}}_{\mathrm{oB}}\right)+\mathrm{c}\mathrm{o}\mathrm{v}\left({\mathrm{a}}_{\mathrm{oA}},{{\mathrm{a}}_{\mathrm{mB}}}_{{}_{\mathrm{D}}}\right)\\ {}+\mathrm{c}\mathrm{o}\mathrm{v}\left({{\mathrm{a}}_{\mathrm{mA}}}_{{}_{\mathrm{D}}},{\mathrm{a}}_{\mathrm{oB}}\right)+\mathrm{c}\mathrm{o}\mathrm{v}\left({{\mathrm{a}}_{\mathrm{mA}}}_{{}_{\mathrm{D}}},{{\mathrm{a}}_{\mathrm{mB}}}_{{}_{\mathrm{D}}}\right).\end{array} $$

Now, we will use the standard regression of an individual’s breeding value on half the breeding values of its parents plus a residual due to Mendelian segregation [49], i.e.

$$ {a}_{oX}=\frac{1}{2}{a}_{o{X}_S}+\frac{1}{2}{a}_{o{X}_D}+{\phi}_{oX}, $$

to expand all the covariance terms to the parental generation. Replacing first the direct breeding value of individual *B* and working out the resulting expression, the first term in the right hand side can be expressed as:

$$ \begin{array}{c}\mathrm{c}\mathrm{o}\mathrm{v}\left({\mathrm{a}}_{\mathrm{o}\mathrm{A}},{\mathrm{a}}_{\mathrm{o}\mathrm{B}}\right)=\frac{1}{2}\mathrm{c}\mathrm{o}\mathrm{v}\left({\mathrm{a}}_{\mathrm{o}\mathrm{A}},{\mathrm{a}}_{\mathrm{o}{\mathrm{B}}_{\mathrm{S}}}\right)+\frac{1}{2}\mathrm{c}\mathrm{o}\mathrm{v}\left({\mathrm{a}}_{\mathrm{o}\mathrm{A}},{{\mathrm{a}}_{\mathrm{o}\mathrm{B}}}_{{}_{\mathrm{D}}}\right)\\ {}+\mathrm{c}\mathrm{o}\mathrm{v}\left({\mathrm{a}}_{\mathrm{o}\mathrm{A}},{\phi}_{\mathrm{o}\mathrm{B}}\right).\end{array} $$

A further step by replacing the breeding value of individual *A*, results in:

$$ \begin{array}{c}\mathrm{c}\mathrm{o}\mathrm{v}\left({\mathrm{a}}_{\mathrm{o}\mathrm{A}},{\mathrm{a}}_{\mathrm{o}\mathrm{B}}\right)=\frac{1}{4}\mathrm{c}\mathrm{o}\mathrm{v}\left({\mathrm{a}}_{\mathrm{o}{\mathrm{A}}_{\mathrm{S}}},{\mathrm{a}}_{\mathrm{o}{\mathrm{B}}_{\mathrm{S}}}\right)+\frac{1}{4}\mathrm{c}\mathrm{o}\mathrm{v}\left({{\mathrm{a}}_{\mathrm{o}\mathrm{A}}}_{{}_{\mathrm{D}}},{{\mathrm{a}}_{\mathrm{o}\mathrm{B}}}_{{}_{\mathrm{S}}}\right)\\ {}+\frac{1}{2}\mathrm{c}\mathrm{o}\mathrm{v}\left({\phi}_{\mathrm{o}\mathrm{A}},{{\mathrm{a}}_{\mathrm{o}\mathrm{B}}}_{{}_S}\right)+\frac{1}{4}\operatorname{cov}\left({\mathrm{a}}_{\mathrm{o}{\mathrm{A}}_{\mathrm{S}}},{{\mathrm{a}}_{\mathrm{o}\mathrm{B}}}_{{}_{\mathrm{D}}}\right)\\ {}+\frac{1}{4}\operatorname{cov}\left({\mathrm{a}}_{\mathrm{o}{\mathrm{A}}_{\mathrm{D}}},{\mathrm{a}}_{\mathrm{o}{\mathrm{B}}_{\mathrm{D}}}\right)+\frac{1}{2}\operatorname{cov}\left({\phi}_{\mathrm{o}\mathrm{A}},{{\mathrm{a}}_{\mathrm{o}\mathrm{B}}}_{{}_{\mathrm{D}}}\right)\\ {}+\frac{1}{2}\operatorname{cov}\left({\mathrm{a}}_{\mathrm{o}{\mathrm{A}}_{\mathrm{S}}},{\phi}_{\mathrm{o}\mathrm{B}}\right)+\frac{1}{2}\operatorname{cov}\left({\mathrm{a}}_{\mathrm{o}{\mathrm{A}}_{\mathrm{D}}},{\phi}_{\mathrm{o}\mathrm{B}}\right)+\operatorname{cov}\left({\phi}_{\mathrm{o}\mathrm{A}},{\phi}_{\mathrm{B}}\right)\end{array} $$

Similarly,

$$ \begin{array}{c}\mathrm{c}\mathrm{o}\mathrm{v}\left({\mathrm{a}}_{\mathrm{o}\mathrm{A}},{\mathrm{a}}_{\mathrm{m}{\mathrm{B}}_{\mathrm{D}}}\right)=\frac{1}{2}\mathrm{c}\mathrm{o}\mathrm{v}\left({{\mathrm{a}}_{\mathrm{o}\mathrm{A}}}_{{}_{\mathrm{S}}},{{\mathrm{a}}_{\mathrm{m}\mathrm{B}}}_{{}_{\mathrm{D}}}\right)+\frac{1}{2}\operatorname{cov}\left({{\mathrm{a}}_{\mathrm{o}\mathrm{A}}}_{{}_{\mathrm{D}}},{{\mathrm{a}}_{\mathrm{m}\mathrm{B}}}_{{}_{\mathrm{D}}}\right)\\ {}+\operatorname{cov}\left({\phi}_{\mathrm{o}{\mathrm{A}}_{\mathrm{S}}},{\mathrm{a}}_{\mathrm{m}{\mathrm{B}}_{\mathrm{D}}}\right),\end{array} $$

and

$$ \begin{array}{c}\mathrm{c}\mathrm{o}\mathrm{v}\left({\mathrm{a}}_{\mathrm{m}{\mathrm{A}}_{\mathrm{D}}},{\mathrm{a}}_{\mathrm{oB}}\right)=\frac{1}{2}\mathrm{c}\mathrm{o}\mathrm{v}\left({\mathrm{a}}_{\mathrm{m}{\mathrm{A}}_{\mathrm{D}}},{{\mathrm{a}}_{\mathrm{oB}}}_{{}_{\mathrm{S}}}\right)+\frac{1}{2}\operatorname{cov}\left({\mathrm{a}}_{\mathrm{m}{\mathrm{A}}_{\mathrm{D}}},{{\mathrm{a}}_{\mathrm{oB}}}_{{}_{\mathrm{D}}}\right)\\ {}+\operatorname{cov}\left({\mathrm{a}}_{\mathrm{m}{\mathrm{A}}_{\mathrm{D}}},{\phi}_{\mathrm{oB}}\right).\end{array} $$

Collecting all these results,

$$ \begin{array}{c}\mathrm{c}\mathrm{o}\mathrm{v}\left({\mathrm{y}}_{\mathrm{A}},{\mathrm{y}}_{\mathrm{B}}\right)=\frac{1}{4}\mathrm{c}\mathrm{o}\mathrm{v}\left({\mathrm{a}}_{\mathrm{o}{\mathrm{A}}_{\mathrm{S}}},{\mathrm{a}}_{\mathrm{o}{\mathrm{B}}_{\mathrm{S}}}\right)+\frac{1}{4}\mathrm{c}\mathrm{o}\mathrm{v}\left({\mathrm{a}}_{\mathrm{o}{\mathrm{A}}_{\mathrm{D}}},{\mathrm{a}}_{\mathrm{o}{\mathrm{B}}_{\mathrm{S}}}\right)\\ {}+\frac{1}{2}\mathrm{c}\mathrm{o}\mathrm{v}\left({\phi}_{\mathrm{o}\mathrm{A}},{\mathrm{a}}_{\mathrm{o}{\mathrm{B}}_{\mathrm{S}}}\right)+\frac{1}{4}\mathrm{c}\mathrm{o}\mathrm{v}\left({\mathrm{a}}_{\mathrm{o}{\mathrm{A}}_{\mathrm{S}}},{\mathrm{a}}_{\mathrm{o}{\mathrm{B}}_{\mathrm{D}}}\right)\\ {}+\frac{1}{4}\mathrm{c}\mathrm{o}\mathrm{v}\left({\mathrm{a}}_{\mathrm{o}{\mathrm{A}}_{\mathrm{D}}},{\mathrm{a}}_{\mathrm{o}{\mathrm{B}}_{\mathrm{D}}}\right)+\frac{1}{2}\mathrm{c}\mathrm{o}\mathrm{v}\left({\phi}_{\mathrm{o}\mathrm{A}},{\mathrm{a}}_{\mathrm{o}{\mathrm{B}}_{\mathrm{D}}}\right)\\ {}+\frac{1}{2}\mathrm{c}\mathrm{o}\mathrm{v}\left({\mathrm{a}}_{\mathrm{o}{\mathrm{A}}_{\mathrm{S}}},{\phi}_{\mathrm{o}\mathrm{B}}\right)+\frac{1}{2}\mathrm{c}\mathrm{o}\mathrm{v}\left({\mathrm{a}}_{\mathrm{o}{\mathrm{A}}_{\mathrm{D}}},{\phi}_{\mathrm{o}\mathrm{B}}\right)\\ {}+\mathrm{c}\mathrm{o}\mathrm{v}\left({\phi}_{\mathrm{o}\mathrm{A}},{\phi}_{\mathrm{o}\mathrm{B}}\right)+\frac{1}{2}\mathrm{c}\mathrm{o}\mathrm{v}\left({\mathrm{a}}_{\mathrm{o}{\mathrm{A}}_{\mathrm{S}}},{\mathrm{a}}_{\mathrm{m}{\mathrm{B}}_{\mathrm{D}}}\right)\\ {}+\frac{1}{2}\mathrm{c}\mathrm{o}\mathrm{v}\left({\mathrm{a}}_{\mathrm{o}{\mathrm{A}}_{\mathrm{D}}},{\mathrm{a}}_{\mathrm{m}{\mathrm{B}}_{\mathrm{D}}}\right)+\mathrm{c}\mathrm{o}\mathrm{v}\left({\phi}_{\mathrm{o}\mathrm{A}},{\mathrm{a}}_{\mathrm{m}{\mathrm{B}}_{\mathrm{D}}}\right)\\ {}+\frac{1}{2}\operatorname{cov}\left({\mathrm{a}}_{\mathrm{m}{\mathrm{A}}_{\mathrm{D}}},{\mathrm{a}}_{\mathrm{o}{\mathrm{B}}_{\mathrm{S}}}\right)+\frac{1}{2}\operatorname{cov}\left({\mathrm{a}}_{\mathrm{m}{\mathrm{A}}_{\mathrm{D}}},{\mathrm{a}}_{\mathrm{o}{\mathrm{B}}_{\mathrm{D}}}\right)\\ {}+\operatorname{cov}\left({\mathrm{a}}_{\mathrm{m}{\mathrm{A}}_{\mathrm{D}}},{\phi}_{\mathrm{o}\mathrm{B}}\right)+\operatorname{cov}\left({\mathrm{a}}_{\mathrm{m}{\mathrm{A}}_{\mathrm{D}}},{\mathrm{a}}_{\mathrm{m}{\mathrm{B}}_{\mathrm{D}}}\right)\end{array} $$

Now, recalling that:

$$ \operatorname{cov}\left({a}_{oX},{a}_{oY}\right)=r\left(X,Y\right){\sigma}_a^2, $$

$$ \operatorname{cov}\left({a}_{oX},{a}_{mY}\right)=r\left(X,{Y}_D\right){\sigma}_{dm}, $$

$$ \operatorname{cov}\left({a}_{mX},{a}_{mY}\right)=r\left({X}_D,{Y}_D\right){\sigma}_m^2, $$

where *r*(*X*,*Y*) stands for the additive relationship between any two individuals *X* and *Y*, the previous expression can be reformulated as:

$$ \begin{array}{c}\operatorname{cov}\left({\mathrm{y}}_{\mathrm{A}},{\mathrm{y}}_{\mathrm{B}}\right)=\frac{1}{4}\mathrm{r}\left({\mathrm{A}}_{\mathrm{S}},{\mathrm{B}}_{\mathrm{S}}\right){\sigma}_d^2+\frac{1}{4}\mathrm{r}\left({\mathrm{A}}_{\mathrm{D}},{\mathrm{B}}_{\mathrm{S}}\right){\sigma}_{\mathrm{d}}^2\\ {}+\frac{1}{2}\operatorname{cov}\left({\phi}_{\mathrm{o}\mathrm{A}},{\mathrm{a}}_{\mathrm{o}{\mathrm{B}}_{\mathrm{s}}}\right)+\frac{1}{4}\mathrm{r}\left({\mathrm{A}}_{\mathrm{S}},{\mathrm{B}}_{\mathrm{D}}\right){\sigma}_{\mathrm{d}}^2\\ {}+\frac{1}{4}\mathrm{r}\left({\mathrm{A}}_{\mathrm{D}},{\mathrm{B}}_{\mathrm{D}}\right){\sigma}_{\mathrm{d}}^2+\frac{1}{2}\operatorname{cov}\left({\phi}_{\mathrm{o}\mathrm{A}},{\mathrm{a}}_{\mathrm{o}{\mathrm{B}}_{\mathrm{D}}}\right)\\ {}+\frac{1}{2}\operatorname{cov}\left({\mathrm{a}}_{\mathrm{o}{\mathrm{A}}_{\mathrm{S}}},{\phi}_{\mathrm{o}\mathrm{B}}\right)+\frac{1}{2}\operatorname{cov}\left({\mathrm{a}}_{\mathrm{o}{\mathrm{A}}_{\mathrm{D}}},{\phi}_{\mathrm{o}\mathrm{B}}\right)\\ {}+\operatorname{cov}\left({\phi}_{\mathrm{o}\mathrm{A}},{\phi}_{\mathrm{o}\mathrm{B}}\right)+\frac{1}{2}\mathrm{r}\left({\mathrm{A}}_{\mathrm{S}},{\mathrm{B}}_{\mathrm{D}}\right){\sigma}_{\mathrm{d}\mathrm{m}}\\ {}+\frac{1}{2}\mathrm{r}\left({\mathrm{A}}_{\mathrm{D}},{\mathrm{B}}_{\mathrm{D}}\right){\sigma}_{\mathrm{d}\mathrm{m}}+\operatorname{cov}\left({\phi}_{\mathrm{o}\mathrm{A}},{\mathrm{a}}_{\mathrm{m}{\mathrm{B}}_{\mathrm{D}}}\right)\\ {}+\frac{1}{2}\mathrm{r}\left({\mathrm{A}}_{\mathrm{D}},{\mathrm{B}}_{\mathrm{S}}\right){\sigma}_{\mathrm{d}\mathrm{m}}+\frac{1}{2}\mathrm{r}\left({\mathrm{A}}_{\mathrm{D}},{\mathrm{B}}_{\mathrm{D}}\right){\sigma}_{\mathrm{d}\mathrm{m}}\\ {}+\operatorname{cov}\left({\mathrm{a}}_{\mathrm{m}{\mathrm{A}}_{\mathrm{D}}},{\phi}_{\mathrm{o}\mathrm{B}}\right)+\frac{1}{2}\mathrm{r}\left({\mathrm{A}}_{\mathrm{D}},{\mathrm{B}}_{\mathrm{D}}\right){\sigma}_{\mathrm{m}}^2\end{array} $$

At this point, we introduce the condition set by Equation (1). Let:

$$ {\sigma}_d^2=-2{\sigma}_{dm}=4{\sigma}_m^2. $$

Then, after proper cancelations and making use of this identity:

$$ \frac{\operatorname{cov}\left({\phi}_{oA},{a}_{o{B}_D}\right)}{\operatorname{cov}\left({\phi}_{oA},{a}_{m{B}_D}\right)}=\frac{\sigma_a^2}{\sigma_{am}}, $$

the expression of the covariance between the two phenotypic values is reduced to the following expression:

$$ \begin{array}{c}\operatorname{cov}\left({\mathrm{y}}_{\mathrm{A}},{\mathrm{y}}_{\mathrm{B}}\right)=\frac{1}{4}\mathrm{r}\left({\mathrm{A}}_{\mathrm{s}},{\mathrm{B}}_{\mathrm{s}}\right){\sigma}_{\mathrm{d}}^2+\frac{1}{2}\operatorname{cov}\left({\phi}_{\mathrm{o}\mathrm{A}},{\mathrm{a}}_{\mathrm{o}{\mathrm{B}}_{\mathrm{S}}}\right)\\ {}+\frac{1}{2}\operatorname{cov}\left({\mathrm{a}}_{\mathrm{o}{\mathrm{A}}_{\mathrm{S}}},{\phi}_{\mathrm{o}\mathrm{B}}\right)+\operatorname{cov}\left({\phi}_{\mathrm{o}\mathrm{A}},{\phi}_{\mathrm{o}\mathrm{B}}\right).\end{array} $$

Notice that the $\operatorname{cov}\left({\phi}_{oX},{a}_{o{Y}_S}\right)>0$ only if *X* is an ancestor of *Y* through the paternal line. In turn, the cov(*ϕ*_*oX*_, *ϕ*_*oY*_) > 0 only if *X* = *Y*. Thus, for any other two individuals in the population:

$$ \operatorname{cov}\left({y}_A,{y}_B\right)=\frac{1}{4}r\left({A}_S,{B}_S\right){\sigma}_d^2. $$

## Additional file

Additional file 1: Table S1. (Co) variance components posterior means (and posterior standard deviations) for the first case of simulation (*σ*_*a*_^2^ = 500, *σ*_*e*_^2^ = 1000) using the genealogical and phenotypic information of the Pirenaica dataset. *σ*_*a*_^2^ and *σ*_*m*_^2^ are the direct and maternal additive genetic variances and *σ*_*am*_ is the direct-maternal genetic covariance. *σ*_*s*_^2^ and *σ*_*d*_^2^ are the paternal and maternal gametic variances and *σ*_*sd*_ is the covariance between the paternal and maternal gametic effects. *σ*_*h*_^2^ and *σ*_*p*_^2^ are the herd and maternal permanent environmental variances. *σ*_*e*_^2^ is the residual variance. **Table S2.** (Co) variance components posterior means (and posterior standard deviations) for the first case of simulation (*σ*_*s*_^2^ = 250, *σ*_*e*_^2^ = 1000) using the genealogical and data structure of the Pirenaica dataset. Description: same as for Table S1. **Table S3.** (Co) variance components posterior means (and posterior standard deviations) for the first case of simulation (*σ*_*a*_^2^ =500, *σ*_*s*_^2^ =250, *σ*_*e*_^2^ =1000) using the genealogical and data structure of the Pirenaica dataset. Description: same as for Table S1. **Table S4.** (Co) variance components posterior means (and posterior standard deviations) for the first case of simulation (*σ*_*a*_^2^ = 500, *σ*_*m*_^2^ = 250, **= −**250, *σ*_*e*_^2^ = 1000) using the genealogical and data structure of the Pirenaica dataset. Description: same as for Table S1. **Table S5.** (Co) variance components posterior means (and posterior standard deviations) for the first case of simulation (*σ*_*a*_^2^ =500, *σ*_*m*_^2^ = 250, *σ*_*am*_ **= −**250, *σ*_*s*_^2^ = 250, *σ*_*e*_^2^ = 1000) using the genealogical and data structure of the Pirenaica dataset. Description: same as for Table S1. (DOCX 168 kb)
