# Supplementary material for: Consequences of paternally inherited effects on the genetic evaluation of maternal effects
Source: Genet Sel Evol. 2015 Aug 13;47(1):63. doi: 10.1186/s12711-015-0141-5 (PMC4534045; doi:10.1186/s12711-015-0141-5)
Supplement: Additional file 1: Table S1. — (Co) variance components posterior means (and posterior standard deviations) for the first case of simulation (σ a2 = 500, σ e2 = 1000) using the genealogical and phenotypic information of the Pirenaica dataset. σ a2 and σ m2 are the direct and maternal additive genetic variances and σ am is the direct-maternal genetic covariance. σ s2 and σ d2 are the paternal and maternal gametic variances and σ sd is the covariance between the paternal and maternal gametic effects. σ h2 and σ p2 are the herd and maternal permanent environmental variances. σ e2 is the residual variance. Table S2. (Co) variance components posterior means (and posterior standard deviations) for the first case of simulation (σ s2 = 250, σ e2 = 1000) using the genealogical and data structure of the Pirenaica dataset. Description: same as for Table S1. Table S3. (Co) variance components posterior means (and posterior standard deviations) for the first case of simulation (σ a2 =500, σ s2 =250, σ e2 =1000) using the genealogical and data structure of the Pirenaica dataset. Description: same as for Table S1. Table S4. (Co) variance components posterior means (and posterior standard deviations) for the first case of simulation (σ a2 = 500, σ m2 = 250, = −250, σ e2 = 1000) using the genealogical and data structure of the Pirenaica dataset. Description: same as for Table S1. Table S5. (Co) variance components posterior means (and posterior standard deviations) for the first case of simulation (σ a2 =500, σ m2 = 250, σ am = −250, σ s2 = 250, σ e2 = 1000) using the genealogical and data structure of the Pirenaica dataset. Description: same as for Table S1. (DOCX 168 kb) [file 12711_2015_141_MOESM1_ESM.docx]

**Table S1 (Co) variance components posterior means (and posterior standard deviations) for Scenario 1 ($\sigma_{a}^{2}$ = 500, $\sigma_{e}^{2}$ = 1000) using the genealogical and phenotypic information of the Pirenaica dataset**

| **Parameter** | **Model of estimation** | | | | | |
| --- | --- | --- | --- | --- | --- | --- |
|  | **A** | **S** | **AS** | **SD** | **AM** | **AMS** |
| $\sigma_{a}^{2}$ | 470.3 (33.7) | - | 427.9 (34.2) | - | 467.4 (40.6) | 368.1(39.2) |
| $\sigma_{m}^{2}$ | - | - | - | - | 26.28 (4.47) | 39.2 (2.5) |
| $\sigma_{am}$ | - | - | - | - | -16.9 (18.6) | 16.9(13.9) |
| $\sigma_{s}^{2}$ | - | 300.9 (28.0) | 43.3 (7.0) | 270.5 (26.1) | - | 59.1(9.6) |
| $\sigma_{d}^{2}$ | - | - | - | 241.2 (14.4) | - | . |
| $\sigma_{sd}$ | - | - | - | 253.3 (11.8) | - | - |
| $\sigma_{h}^{2}$ | 4.8 (3.9) | 6.3 (4.7) | 4.5 (3.8) | 4.4 (3.7) | 4.6 (3.8) | 4.5 (3.8) |
| $\sigma_{p}^{2}$ | 11.4 (6.0) | 110.6 (13.1) | 12.4 (6.4) | 9.2 (4.9) | 9.5 (5.1) | 8.7 (4.8) |
| $\sigma_{e}^{2}$ | 997.9 (24.4) | 1096.8 (23.1) | 990.9 (24.3) | 972.2 (24.4) | 994.6 (25.8) | 1012.0 (24.0) |

$\sigma_{a}^{2}$ and $\sigma_{m}^{2}$ are the direct and maternal additive genetic variances and $\sigma_{am}$ is the direct-maternal genetic covariance. $\sigma_{s}^{2}$ and $\sigma_{d}^{2}$ are the paternal and maternal gametic variances and $\sigma_{sd}$ is the covariance between the paternal and maternal gametic effects. $\sigma_{h}^{2}$ and $\sigma_{p}^{2}$ are the herd and maternal permanent environmental variances. $\sigma_{e}^{2}$ is the residual variance.

**Table S2** (**Co) variance components posterior means (and posterior standard deviations) for Scenario 2 ($\sigma_{s}^{2}$ = 250, $\sigma_{e}^{2}$ = 1000) using the genealogical and data structure of the Pirenaica dataset**

| **Parameter** | **Model of estimation** | | | | | |
| --- | --- | --- | --- | --- | --- | --- |
|  | **A** | **S** | **AS** | **SD** | **AM** | **AMS** |
| $\sigma_{a}^{2}$ | 209.2 (17.2) | - | 23.5 (3.1) | - | 414.8 (18.3) | 124.3 (8.4) |
| $\sigma_{m}^{2}$ | - | - | - | - | 116.6 (6.3) | 38.8 (3.9) |
| $\sigma_{am}$ | - | - | - | - | -211.6 (9.7) | -60.9 (5.1) |
| $\sigma_{s}^{2}$ | - | 220.8 (23.6) | 205.0 (21.5) | 215.8 (24.6) | - | 146.5 (21.1) |
| $\sigma_{d}^{2}$ | - | - | - | 14.3 (6.9) | - | - |
| $\sigma_{sd}$ | - | - | - | 22.9 (5.0) | - | - |
| $\sigma_{h}^{2}$ | 5.2 (4.0) | 2.4 (2.5) | 2.4 (2.5) | 2.5 (2.5) | 2.4 (2.4) | 2.5 (2.5) |
| $\sigma_{p}^{2}$ | 4.0 (2.3) | 5.9 (3.3) | 5.2 (2.9) | 4.6 (2.7) | 4.8 (2.7) | 4.8 (2.7) |
| $\sigma_{e}^{2}$ | 1013.8 (16.5) | 1019.4 (18.4) | 1011.4 (17.2) | 1003.5 (20.7) | 915.4 (15.5) | 987.9 (17.7) |

$\sigma_{a}^{2}$ and $\sigma_{m}^{2}$ are the direct and maternal additive genetic variances and $\sigma_{am}$ is the direct-maternal genetic covariance. $\sigma_{s}^{2}$ and $\sigma_{d}^{2}$ are the paternal and maternal gametic variances and $\sigma_{sd}$ is the covariance between the paternal and maternal gametic effects. $\sigma_{h}^{2}$ and $\sigma_{p}^{2}$ are the herd and maternal permanent environmental variances. $\sigma_{e}^{2}$ is the residual variance.

**Table S3 (Co) variance components posterior means (and posterior standard deviations) for Scenario 3 ($\sigma_{a}^{2}$=500, $\sigma_{s}^{2}$=250, $\sigma_{e}^{2}$=1000) using the genealogical and data structure of the Pirenaica dataset**

| **Parameter** | **Model of estimation** | | | | | |
| --- | --- | --- | --- | --- | --- | --- |
|  | **A** | **S** | **AS** | **SD** | **AM** | **AMS** |
| $\sigma_{a}^{2}$ | 677.6 (38.3) | - | 475.8 (53.5) | - | 943.9 (66.7) | 458.7 (99.3) |
| $\sigma_{m}^{2}$ | - | - | - | - | 144.7 (19.4) | 47.1 (13.8) |
| $\sigma_{am}$ | - | - | - | - | -256.5 (33.2) | -27.2 (27.4) |
| $\sigma_{s}^{2}$ | - | 564.6 (47.4) | 285.3 (46.1) | 512.8 (47.0) | - | 252.5 (57.1) |
| $\sigma_{d}^{2}$ | - | - | - | 246.4 (27.4) | - | - |
| $\sigma_{sd}$ | - | - | - | 250.8 (18.1) | - | - |
| $\sigma_{h}^{2}$ | 8.7 (5.2) | 7.6 (5.0) | 5.3 (4.2) | 5.3 (4.4) | 5.8 (4.3) | 7.1 (6.7) |
| $\sigma_{p}^{2}$ | 6.5 (3.9) | 106.8 (14.1) | 9.4 (5.6) | 8.0 (4.5) | 8.7 (4.8) | 8.1 (10.8) |
| $\sigma_{e}^{2}$ | 991.5 (27.6) | 1065.9 (30.9) | 952.3 (27.5) | 955.5 (33.5) | 915.4 (15.5) | 968.6 (57.1) |

$\sigma_{a}^{2}$ and $\sigma_{m}^{2}$ are the direct and maternal additive genetic variances and $\sigma_{am}$ is the direct-maternal genetic covariance. $\sigma_{s}^{2}$ and $\sigma_{d}^{2}$ are the paternal and maternal gametic variances and $\sigma_{sd}$ is the covariance between the paternal and maternal gametic effects. $\sigma_{h}^{2}$ and $\sigma_{p}^{2}$ are the herd and maternal permanent environmental variances. $\sigma_{e}^{2}$ is the residual variance.

**Table S4 (Co) variance components posterior means (and posterior standard deviations) for Scenario 4 ($\sigma_{a}^{2}$ = 500, $\sigma_{m}^{2}$ = 250, $\sigma_{am}$ = -250, $\sigma_{e}^{2}$ = 1000) using the genealogical and data structure of the Pirenaica dataset**

| **Parameter** | **Model of estimation** | | | | | |
| --- | --- | --- | --- | --- | --- | --- |
|  | **A** | **S** | **AS** | **SD** | **AM** | **AMS** |
| $\sigma_{a}^{2}$ | 353.1 (45.7) | - | 207.1 (32.3) | - | 475.4 (81.7) | 166.1 (24.32) |
| $\sigma_{m}^{2}$ | - | - | - | - | 246.5 (53.8) | 158.1 (38.3) |
| $\sigma_{am}$ | - | - | - | - | -246.8 (68.3) | -82.8 (38.5) |
| $\sigma_{s}^{2}$ | - | 273.5 (45.8) | 199.2 (39.3) | 257.1 (46.1) | - | 170.1 (29.7) |
| $\sigma_{d}^{2}$ | - | - | - | 185.0 ( 46.1) | - | - |
| $\sigma_{sd}$ | - | - | - | -0.6 (46.1) | - | - |
| $\sigma_{h}^{2}$ | 13.4 (6.9) | 9.3 (6.4) | 7.2 (5.7) | 6.9 (5.9) | 7.0 (5.9) | 6.5 (5.5) |
| $\sigma_{p}^{2}$ | 48.4 (26.6) | 109.1 (45.8) | 53.2 (16.0) | 26.9 (19.0) | 15.1 (12.0) | 15.5 (12.0) |
| $\sigma_{e}^{2}$ | 1080.9 (28.6) | 1114.4 (30.7) | 1047.8 (32.2) | 1026.8 (46.7) | 998.2 (45.4) | 1068.9 (29.5) |

$\sigma_{a}^{2}$ and $\sigma_{m}^{2}$ are the direct and maternal additive genetic variances and $\sigma_{am}$ is the direct-maternal genetic covariance. $\sigma_{s}^{2}$ and $\sigma_{d}^{2}$ are the paternal and maternal gametic variances and $\sigma_{sd}$ is the covariance between the paternal and maternal gametic effects. $\sigma_{h}^{2}$ and $\sigma_{p}^{2}$ are the herd and maternal permanent environmental variances. $\sigma_{e}^{2}$ is the residual variance.

**Table S5 (Co) variance components posterior means (and posterior standard deviations) for Scenario 5 ($\sigma_{a}^{2}$ = 500, $\sigma_{m}^{2}$ = 250, $\sigma_{am}$ = -250, $\sigma_{s}^{2}$ = 250, $\sigma_{e}^{2}$ = 1000) using the genealogical and data structure of the Pirenaica dataset**

| **Parameter** | **Model of estimation** | | | | | |
| --- | --- | --- | --- | --- | --- | --- |
|  | **A** | **S** | **AS** | **SD** | **AM** | **AMS** |
| $\sigma_{a}^{2}$ | 541.61 (73.29) | - | 349.39 (70.05) | - | 947.85 (163.38) | 529.30 (338.45) |
| $\sigma_{m}^{2}$ | - | - | - | - | 331.71 (84.39) | 270.05 (112.69) |
| $\sigma_{am}$ | - | - | - | - | -470.20 (111.86) | -253.92 (189.71) |
| $\sigma_{s}^{2}$ | - | 577.59 (66.34) | 381.32 (97.57) | 523.83 (90.54) | - | 258.45 (168.42) |
| $\sigma_{d}^{2}$ | - | - | - | 256.92 (78.00) | - | - |
| $\sigma_{sd}$ | - | - | - | -35.99 (53.41) | - | - |
| $\sigma_{h}^{2}$ | 18.50 (10.54) | 8.19 (6.50) | 6.51 (6.99) | 6.70 (7.64) | 6.77 (7.73) | 6.12 (6.60) |
| $\sigma_{p}^{2}$ | 66.85 (23.02) | 155.24 (19.93) | 57.96 (24.64) | 31.35 (31.90) | 19.24 (19.52) | 19.19 (19.00) |
| $\sigma_{e}^{2}$ | 1167.08 (45.07) | 1185.11 (41.23) | 1013.52 (64.70) | 990.24 (76.16) | 842.51 (84.49) | 972.35 (108.64) |

$\sigma_{a}^{2}$ and $\sigma_{m}^{2}$ are the direct and maternal additive genetic variances and $\sigma_{am}$ is the direct-maternal genetic covariance. $\sigma_{s}^{2}$ and $\sigma_{d}^{2}$ are the paternal and maternal gametic variances and $\sigma_{sd}$ is the covariance between the paternal and maternal gametic effects. $\sigma_{h}^{2}$ and $\sigma_{p}^{2}$ are the herd and maternal permanent environmental variances. $\sigma_{e}^{2}$ is the residual variance.
